# How late is too late for nerve transfers

**DOI:** 10.1186/1753-6561-9-S3-A20

**Published:** 2015-05-19

**Authors:** Anil Bhatia

**Affiliations:** 1 Joshi Hospital, Pune, Maharashtra 411004, India

## 

Traditionally, the degeneration of the motor endplates has placed a limit on the accepted delay for nerve reconstruction. Nerve transfers have helped to deliver growing axons very close to the target muscles so that the post-operative denervation is shortened. Despite efforts at spreading awareness of surgical indications, there are instances where the patient presents late to a specialized brachial plexus unit. In such cases, one faces the dilemma of using traditional nerve transfers or opting for a free functioning muscle transfer. Some surgeons rely on the presence of fibrillations on the EMG to help them decide about the feasibility of re-innervation. However, there are no clear guidelines.

I would like to present a study of 50 patients operated at 9 months or later (9-19.5 months) from the accident. I have excluded cases operated later than April 2013, as the follow-up is too short. Their ages ranged from 4-57 years with a mean of 28.6 years (31 of the 50 patients belonged the range 10-30 years). There were 15 total palsies, 15 cases of C567 palsies, 9 cases of C56 palsies, one case of isolated C8T1 involvement and 10 infraclavicular palsies (injuries beyond the trunks).

Among the nerve transfers for elbow flexion, intercostals were transferred to the musculocutaneous in 15 cases. Biceps of at least grade 3 strength was restored in 5/15 patients. Ulnar to biceps transfer was done in 22 cases (+median to brachialis in 16 of these). 17 of these patients regained elbow flexion stronger than grade 3. The spinal accessory nerve was transferred to the suprascapular nerve in 34 cases and grade 3 supraspinatus was achieved in 20 patients. However, the cases of successful restoration of shoulder abduction included 7 patients with C56 palsies and 4 of C567 palsies. Thus, from these observations it appears that:

1. In total palsies, the failure rate of intercostals to musculocutaneous and spinal accessory to suprascular nerve transfers is far higher than the overall average in my series.

2. The failure rate for ulnar to biceps transfer remains unchanged even up to 19.5 months from the accident.

3. Shoulder abduction is a reasonable expectation after a nerve transfer in a partial palsy even at delays longer than 10 months.

I recommend that one should not succumb to the temptation to perform traditional nerve reconstruction in patients with flail upper limbs that present late, even if they are young. The available nerve transfers should be utilized for innervation of free functioning muscles. On the other hand, we can continue to have an optimistic outlook for C56 palsies even at delays as long as 18-19 months.

